# T Cell Deficits and Overexpression of Hepatocyte Growth Factor in Anti-inflammatory Circulating Monocytes of Middle-Aged Patients with Bipolar Disorder Characterized by a High Prevalence of the Metabolic Syndrome

**DOI:** 10.3389/fpsyt.2017.00034

**Published:** 2017-03-20

**Authors:** Rogier J. Vogels, Manja A. Koenders, Elisabeth F. C. van Rossum, Annet T. Spijker, Hemmo A. Drexhage

**Affiliations:** ^1^Department of Immunology, Erasmus Medical Center, Rotterdam, Netherlands; ^2^Department of Mood Disorders, PsyQ, Rotterdam, Netherlands; ^3^Division of Endocrinology, Department of Internal Medicine, Erasmus Medical Center, Rotterdam, Netherlands; ^4^Obesity Center CGG, Rotterdam, Netherlands

**Keywords:** bipolar disorder, T cell deficits, angiogenic cells, gene expression, hypothalamic–pituitary–adrenal axis

## Abstract

**Background:**

We previously reported T cell deficits and pro-inflammatory gene activation in circulating monocytes of two cohorts of bipolar disorder (BD) patients, a cohort of postpartum psychosis patients and in bipolar offspring. Pro-inflammatory gene activation occurred in two clusters of mutually correlating genes, cluster 1 for inflammation-related cytokines/factors, cluster 2 for motility, chemotaxis, and metabolic factors.

**Aim:**

To verify these cellular immune abnormalities in yet another cohort [the bipolar stress study (BiSS) cohort] of relative old (52 years, median) BD patients and to relate immune abnormalities to hair cortisol levels, measured in this cohort and representing long-term systemic cortisol levels, and to the presence of the metabolic syndrome (MetS), which was prevalent in 29% of the BiSS patients.

**Methods:**

Monocyte immune gene activation (quantitative polymerase chain reaction) and T cell deficits (fluorescence-activated cell sorting analysis) were determined in 97 well-controlled, largely euthymic BiSS BD patients. Monocyte genes included the cluster 1 and 2 genes, the genes for the glucocorticoid receptor (GR) α and GRβ, and the gene for hepatocyte growth factor [HGF, a marker of monocyte-derived circulating angiogenic cells (CACs)]. CACs serve vessel repair. Abnormal numbers are found in patients with MetS and vascular damage.

**Results:**

As compared to healthy controls: (1) the pro-inflammatory cluster 1 genes were downregulated, and the GRα and the HGF gene were upregulated in the monocytes of the BiSS patients and (2) T cell deficits were shown (reduced numbers of lymphocytes in particular of T cells). Within the reduced T cell population, a shift had taken place in the T-helper populations: T-helper 17 and T-helper 2 increased and T regulatory cells decreased. Correlations between hair cortisol, the MetS, monocyte gene activation, and T cell deficits were not found.

**Conclusion:**

T cell deficits most likely are a trait phenomenon of BD, since they have also been found in the other cohorts of BD patients and in bipolar offspring. Monocytes of this cohort showed an anti-inflammatory set point, suggesting that pro- and anti-inflammation are state characteristics of BD. The monocyte gene profile indicated an increased CAC activity; the question arises whether this is due to putative vessel damage in these relatively old patients with a high prevalence of the MetS.

## Introduction

The last 10 years evidence has accumulated that dysfunctions of T cells, and monocytes/macrophages are important factors in the development of bipolar disorder (BD). The majority of studies focused on cytokine levels in serum, and a recent meta-analysis confirmed the elevation of pro-inflammatory, anti-inflammatory, and regulatory cytokines in BD (1).

Cellular studies have accumulated too. Our team has studied in the past 10 years the percentages of circulating subsets of T cells and the monocyte inflammatory gene expression in three patient cohorts of BD patients. We have studied (1) a cohort of 56 BD patients from the Dutch site of the Stanley Foundation Bipolar Network (D-SFBN) cohort, mean age 42 years (2, 3), (2) a cohort of 90 BD patients, aged 43 years (mean) from the MOODINFLAME-Groningen-Leuven site (4), and (3) a cohort of 140 children of a bipolar parent in follow-up, from 16 through 29 years, the so-called Dutch bipolar offspring (DBO) study (5). In these studies, we detected that the monocytes of subjects were characterized by the abnormal expression of two coherent clusters of inflammation-related genes. One cluster of monocyte genes represented pro-inflammatory cytokines and key regulating compounds for the production of these cytokines (cluster 1 with genes, such as IL1B, IL6, TNF, PDE4B, and ATF3). The other cluster represents monocyte genes playing a role in chemotaxis, motility, nuclear signaling, and metabolism (cluster 2 with genes, such as CCL2, CCL7, NAB2, PTPN7, and DHRS3). The two clusters were found upregulated in monocytes particularly during a manic/depressive episode (4, 6), in longstanding disease with an early onset (6), and in 16-year-old DBO subject irrespective of the presence of mood symptoms (5).

The pro-inflammatory state of the monocytes was also found in yet another cohort of patients (a fourth cohort), namely, patients with postpartum psychosis, using not only gene expression but also microRNA profiling (7, 8). Postpartum psychosis is a pathologic condition related to BD. In postpartum disorder, monocytes not only showed increased levels of cluster 1 and 2 genes (7) but also decreased levels of miR-146a (8). MiR-146a is a well-established inflammation-regulating microRNA, and its decrease underscored the pro-inflammatory state of the patient monocytes. In addition, *in silico* studies showed monocyte miR-146a to target genes of the pro-inflammatory monocyte signature, such as PTGS2 and IRAK2.

Indeed, microRNA studies form an entire new field to investigate a new layer of epigenetic regulation of cellular function. While the study of Weigelt et al. (8) and those of others (9) target circulating leukocytes, most studies in psychiatry have focused on abnormal microRNA expression in the brain investigating microRNAs playing a role in brain development [reviewed in Ref. (10)].

Apart from the pro-inflammatory state of monocytes, we also detected in these four cohort studies that the T cell system was set at another equilibrium. In all cohorts, there was a deficit in the number of T cells, while within the T cell population there were abnormal fluctuations over time in the circulating levels of anti-inflammatory T regulatory (Treg) cells and T-helper 2 (Th2) cells and of pro-inflammatory T-helper 1 (Th1) and T-helper 17 (Th17) cells (7, 11, 12).

These observations enforced our idea that the immune system of individuals at high risk to develop BD (the DBO subjects) and of BD patients was abnormal and dynamically activated and deactivated over time showing episodes of inflammatory activation, particularly in early stages of the disease process in adolescence and the absence of mood symptoms and during active disease.

Recently, we collected circulating leukocytes of another cohort of BD patients. These patients belonged to the so-called Bipolar Stress Study (BiSS) cohort and had a mean age of 52 years (significantly older than the Stanley Foundation, the MOODINFLAME, and the DBO cohort). The BiSS study is a 2-year longitudinal study, designed to identify risk factors that have an impact on the clinical course and the treatment of outpatients with BD (13). The cohort has been studied in depth clinically (14–17) and is special in that the subjects had also been studied for the presence of the metabolic syndrome (MetS) (18). BiSS subjects showed a significantly higher prevalence of MetS when compared to subjects with MDD and non-psychiatric controls of the same age (28.4 versus 20.2 and 16.5%, respectively, *p* < 0.001), also when adjusted for sociodemographic and lifestyle factors. It is known that patients with severe mental illness do have a higher risk for cardiovascular disease (19).

Also special is that BiSS subjects had been studied for their hypothalamic–pituitary–adrenal (HPA) axis activity over a longer period of time *via* measuring their hair cortisol level (16). The hair cortisol test is relatively novel and reflects the mean HPA-axis activity over a period of time, in our case over the last 3 months before the leukocyte collection. Staufenbiel et al. (17) reported an increase of hair cortisol levels in the BD patients of the BiSS cohort, but only in those with negative life events within 3 months before the cortisol determination. Hair cortisol was found normal across the entire BiSS cohort.

The MetS, the HPA axis, and the immune system have strong mutual influences (20–22). The collection of circulating leukocytes in the BiSS cohort at the end of the 2-year follow-up period enabled us to study the monocyte gene expression and T cell subset distribution in the BiSS subjects in relation to the mean HPA-axis activity in the 3-month period before blood collection and in relation to the presence/absence of the MetS.

We therefore not only determined the earlier reported cluster 1 and 2 gene sets in the monocytes of the BiSS subject but also the gene expression levels of the genes for the glucocorticoid receptor α (GRα, activating) and GRβ (blocking receptor). We hypothesized that there would a relationship between the expression of the GR receptor in monocytes, the cluster 1 and 2 gene expression, and the hair cortisol levels.

Not only did we add the genes for the GR but also additionally determined the expression of the gene for hepatocyte growth factor (HGF). In a recent study on type 2 diabetic (T2D) and MetS patients (23), we found HGF gene expression raised in the circulating monocytes of the T2D patients. HGF is an important vascular repair factor (24) and monocyte-derived circulating angiogenic cells (CACs), capable of repairing endothelial damage, are characterized by the expression of HGF (25). We hypothesized that the HGF expression would be raised in particularly BiSS subjects with the MetS.

In addition to monocyte gene expression studies, fluorescence-activated cell sorting (FACS) analysis was performed on the collected leukocytes of the BiSS cohort to determine the percentages of monocytes, lymphocytes, B cells, NK cells, T cells, as well as the distribution of the T cells into the cytotoxic T cells and the helper T cells. The latter population was further quantified for its subpopulations, i.e., the regulatory T cells, the Th1 cells, the Th2 cells, and the Th17 cells. We hypothesized that we would detect again the T cell deficits and an abnormal distribution of T-helper subsets within the circulating T cell population of the BiSS subjects, similar as was found before.

Thus, our prime objective of this study was to determine the monocyte gene activation patterns and leukocyte distribution abnormalities in this new BiSS cohort of BD patients, and to compare outcomes to the outcomes of the previous studied BD cohorts, which had been investigated with the same techniques. Our secondary objective was to study correlations between the immune outcomes and the hair cortisol level and the presence/absence of the MetS.

## Materials and Methods

### Patients

The study design and patient population have been described elsewhere in detail (13–15, 17). For this study, only patients with hair cortisol measurements were selected. The study was conducted as part of the MOODINFLAME (EU-FP7-HEALTH-F2-2008-222963) and the PSYCHAID project (EU-FP7-PEOPLE-2009-IAPP-MarieCurie-286334) on the samples cross-sectional collected at the end of the 2-year follow-up study. The study protocol was approved by the Medical Ethics Committee of the Clinic for Mood disorders, Parnassia Group (presently PsyQ), The Hague, The Netherlands, and all patients gave informed consent after a full description of the study.

To summarize, adult patients (*n* = 97) with BD I, II, and not otherwise specified were included. As this was a naturalistic study design, we included all outpatients with BD. Diagnosis of BD and psychiatric comorbidities were based on DSM-IV criteria and were assessed with the MINI International Neuropsychiatric Interview Plus (26). Sociodemographic data and disease-related data were collected in interviews by trained psychologists. Disease-related data included disease characteristics (age of onset, disease duration, mood classification, comorbidities including the MetS) and medication use (lithium, antipsychotics, anti-depressives, and benzodiazepines). Mood classification was administrated with the self-rated Quick Inventory of Depressive Symptomatology and the observer based Young Mania Rating Scale (27, 28). MetS was defined according to the National Cholesterol Education program—Adult Treatment panel III (29).

Exclusion criteria were age below 18 years and the diagnosis schizoaffective disorder. As the BiSS study was a naturalistic study, we included all patients with BD and only noted comorbidities, such as thyroid disease, diabetes, and endocrine disease in general. Patients with glucocorticoid usage in the last 6 months or without sufficient hair growth at the posterior vertex were also excluded (this was interfering with the hair cortisol determination). Methods and detailed data on the hair cortisol levels and GR polymorphisms are available in previous studies on this cohort (15–17). The reference upper limit of the normal hair cortisol level was defined by Manenschijn et al. (30) as 52 pg/mg hair in healthy non-obese individuals. The reference lower limit has not yet been determined.

### Healthy Controls (HCs)

The HC group was established by combining HCs of the studies in the MOODINFLAME and PSYCHAID consortia (Departments of Psychiatry, University Hospitals of Rotterdam, Groningen, Münster, Leuven, and München). These HCs had been selected at the same time as the patients, and material was processed in the same way by the same technicians together with the materials of the patients. Exclusion criteria for HC in the MOODINFLAME and PSYCHAID studies were psychiatric and immune disease or endocrine disease in both the subject and first-degree family members and medication use (apart from oral contraceptives). Of a total number of 519 HC with peripheral blood mononuclear cell (PBMC) data available, only HC with complete information on gender, age, and BMI was included for comparison (*n* = 272). From these 272 controls, we selected 47 controls for the monocyte gene expression evaluation, since these controls had been tested in the same assays as the BD cases (to avoid inter-assay variation). For the leukocyte subset determination, we finally selected 72 controls matched for age and gender (see Results).

### Blood Collection and Preparation

Blood was drawn using sodium heparin tubes for immune cell preparation, and the heparinized blood was subsequently centrifuged to prepare PBMC suspensions as described previously in detail (7, 31). PBMCs were frozen in 10% dimethyl sulfoxide and stored in liquid nitrogen.

### Determination of Monocyte Gene Expression

CD14^+^ monocytes were isolated from the frozen PBMCs using a magnetic cell sorting system (Miltenyi Biotec, Germany) resulting in a purity of monocytes >95%. RNA was isolated from the purified monocytes, and 1 μg of RNA was reverse transcribed using the cDNA high-capacity cDNA Reverse Transcription Kit (Applied Biosystems, Carlsbad, CA, USA). The cDNA was used in quantitative polymerase chain reaction (qPCR) to determine gene expression using the comparative threshold (CT) cycle method (32). qPCR was performed using a TaqMan Universal PCR mastermix and TaqMan probes (Applied Biosystems). Gene expression was normalized to expression of housekeeping gene ABL1, and the resulting ΔCT values were used for further analyses.

Genes of interest were selected based on previous findings of abnormally expressed genes in patients with BD (4, 5, 11), MDD (31), type 2 diabetes (23), and systemic autoimmune diseases [the IFN-induced inflammatory genes (33)], and known to be involved in major inflammatory or immune activation pathways in monocytes and macrophages. We selected the most consistently over expressed genes. A list of included genes is given in Table S1 in Supplementary Material.

### Monocyte Gene Expression Analysis

It has been published extensively before that the determined genes are expressed in three mutually coherent clusters, i.e., cluster 1, cluster 2, and the IFN cluster (7, 11, 23, 31, 33). For this study, principle component analysis was used to determine which genes could be allocated to these clusters. Furthermore, the analysis showed the order of correlation of each gene in the found clusters, enabling the use of a limited set of the highest correlating genes (key genes) for further comparisons. ΔΔCT values (ΔCT values of patients relative to the mean ΔCT value of controls within the same assay) were used to compare gene expression of BD patients with HC for the key genes of each cluster. The results were expressed as fold change (FC) relative to HC. The researcher was blind to the identity of the sample during the FACS measurement and analysis of the data.

### Determination of the Percentage and Subset Distribution of Circulating Leukocytes

Lymphocytes, monocytes (CD14^+^), B cells (CD19^+^), NK cells (CD56^+^), total T cells (CD3^+^), T-helper cells (CD4^+^), cytotoxic T cells (CD8^+^), and regulatory T cells (CD25^+^FoxP3^+^) were determined using FACS and described as percentage relative to total mononuclear cells. Furthermore, Th1 (IFN-γ^+^), Th2 (IL4^+^), and Th17 (IL17A^+^) subsets were assessed and described relative to the total number of leukocytes. All used procedures including the gating strategy are extensively described in Snijders et al. (12). The researcher was blind to the identity of the sample during the FACS measurement and analysis of the data.

### Statistics

Statistical analysis was performed using SPSS software, version 23.0 (IBM Corp, Armonk, NY, USA). Normally distributed continuous variables were expressed as mean with 95% confidence intervals and compared using two-sided *T*-tests. Log transformation was applied on Th1, Th2, and Th17 cells and hair cortisol levels to achieve normality. Non-normal data, mainly the various clinical variables, were described with median (range) and compared with Mann–Whitney *U* tests.

Principle component analysis was used on the 36 genes (excluding HGF and both GR isotypes) with oblique rotation (direct oblimin). The Kaiser–Meyer–Olkin measure verified the sampling adequacy, KMO = 0.89. An initial analysis was run to determine the number of factors to be extracted. A combination of three factors with the highest eigenvalue and clear inflections shown in the scree plot explained 63.31% of the variance. The maximum number of convergence was 25.

For monocyte gene expression one-sample *T*-test was performed on normally distributed ΔΔCT values, and data are presented as FC of the patient gene expression relative to the HC value set to 1 for each given gene.

## Results

### Clinical Characteristics of the Patients, including the Prevalence of the MetS and Hair Cortisol

The large majority of the BD patients were BD I cases (76%), had longstanding disease (mean duration 23 years), were on lithium (76%), were well-controlled with low self-rated QIDS scores (median 5), and showed a high prevalence of the MetS (29%). Further detailed characteristics of BD patients are given in Table 1.

**Table 1:** **Characteristics of bipolar patients and healthy controls**.

	All bipolar disorder patients (*n* = 97)
**Age** (median, range), years	52 (20–83)
**Female** (*n*, %)	61 (63)
**BMI** (mean, SD)	26.2 (4.5)
**DSM-IV classification** (*n*, %)	
Bipolar I	74 (76)
Bipolar II	22 (23)
Non-specified	1 (1)
**QIDS score** (median, range)	5 (0–22)
**Mood classification** (*n*, %)	
Euthymic^a^	50 (52)
Mild depression^b^	23 (24)
Moderate depression^c^	14 (14)
Severe depression^d^	5 (5)
Very severe depression^e^	1 (1)
Manic^f^	0
Not determined	4
**Age of onset** (median, range), years	21.5 (20–59)
**Disease duration** (median, range), years	23 (0–61)
**Metabolic syndrome** (*n*, %)	28 (29)
**Endocrine disease** (*n*, %)	10 (10)
Thyroid disease^g^	7 (7)
Diabetes mellitus type 2	3 (3)
**Smoking** (*n*, %)	32 (33)
**Medication** (*n*, %)	
Lithium	74 (76)
Antipsychotics	25 (26)
Antidepressants	30 (31)
Benzodiazepines	29 (30)

*^a^Self-rated Quick Inventory of Depressive Symptomatology (QIDS-SR) score ≤5, YMRS <13*.

*^b^QIDS-SR score 6–10*.

*^c^QIDS-SR score ≥11*.

*^d^QIDS-SR score ≥16*.

*^e^QIDS-SR score ≥21*.

*^f^Young Mania Rating Scale score ≥13*.

*^g^Stable disease (normal TSH + fT4)*.

Hair cortisol levels of the BiSS cohort have been published in detail before (16, 17). Using the cut-off determined by Staufenbiel et al. (17), the large majority of BD patients showed normal hair cortisol levels (*n* = 84, median = 25.6, range = 10.6–51.9), the remaining patients had elevated cortisol levels (*n* = 13, median = 77.2, range = 56.7–370.8).

### Monocyte Gene Expression

A total of 39 genes were included in qPCR, shown with description in Table S1 in Supplementary Material. Monocyte gene expression could successfully be determined in 97 BD patients. Monocyte gene expression of patients was compared to monocyte gene expression of 47 HC of whom blood was tested in the same assays (to avoid inter-assay variation for monocyte gene expression). Of these 47 healthy subject data on gender, age and BMI were available: median age was 48 years (range 24–72), percentage females 57, and a mean BMI of 25.6 (SD 3.6). BMI and gender were not statistically significantly different between patients and HC. The age of the HC (48 years, mean) was statistically different from the age of the patients (52 years, mean, *p* < 0.01). However, age was not found to correlate to monocyte gene expression (data not shown). Nevertheless, and to be sure, corrections were carried out in statistics for age, gender, and BMI.

The results of the principle component analysis are shown in Table S2 in Supplementary Material. As expected the three distinct clusters could be found as in previous studies (7, 11, 23, 31, 33). The first cluster was by and large corresponding to the previously described cluster 1 (pro-inflammatory genes) and the second by and large to the previously described cluster 2 (genes involved in chemotaxis, cell adhesion nuclear signaling, and motility) using the previous cohorts of BD patients. The principle component analysis also showed a third cluster, i.e., a cluster of interferon-induced genes, on which we have extensively published in systemic autoimmune diseases, such as Sjögren disease, SLE, and systemic sclerosis (33). In these latter diseases, this gene set is over expressed in monocytes due to high levels of type II IFNs in the circulation.

Some of the lower ranking genes of cluster 1 (like HSPA1A/B) and of cluster 2 (like CD9) in the previous studies did not belong to the present clusters in this study anymore (Table S2 in Supplementary Material). Since there is not an exact overlap between the clusters in the various studies, we only took the three top genes of the principle component analysis into consideration for further analysis and calculations. These top genes belong in each of the previous study also to the most discriminative genes for the cluster.

The fold expression of the top three genes per cluster as found with principle component analysis is shown relative to HCs in Table 2 (the outcomes of the entire list of measured genes can be found in Table S3 in Supplementary Material). Gene cluster 1, as measured by the top key genes IL1B, CCL20, and IL6, was significantly downregulated in BD patients, Table S3 in Supplementary Material shows that indeed the vast majority of cluster 1 genes (13 out of 15) is significantly downregulated. We take this observation as indicating that the monocytes of the BiSS BD patients are set at an anti-inflammatory set point.

**Table 2:** **Monocytes in bipolar disorder patients compared to healthy control (HC)**.

	FC^a^	95% CI	*p* Value
**Cluster 1**			
IL1B	**0.72**	0.59–0.89	**0.002**
CCL20	**0.58**	0.43–0.78	**0.001**
IL6	**0.76**	0.61–0.96	**0.021**
**Cluster 2**			
NAB2	**0.80**	0.67–0.96	**0.017**
CCL7	0.93	0.65–1.32	0.669
CCL2	1.05	0.82–1.34	0.703
**Interferon-related cluster**			
IFI44	1.01	0.90–1.13	0.860
IFIT3	1.04	0.90–1.19	0.631
Glucocorticoid receptor (GR) α	**1.03**	1.00–1.07	**0.042**
GRβ	0.97	0.87–1.09	0.626
Hepatocyte growth factor	**1.10**	1.03–1.18	**0.005**

*^a^Fold change (FC) relative to HC*.

*The most important genes for each respective cluster are shown as found by principle component analysis (Table S2 in Supplementary Material)*.

*A full list of compared genes is included in Table S3 in Supplementary Material*.

*Values marked in bold indicate numbers that are significant on the 95% confidence limit*.

Of the cluster 2 key genes, only the top key gene NAB2 showed a significantly reduced expression as compared to the expression level in the monocytes of HCs; Table S3 in Supplementary Material shows that indeed only a minority of cluster 2 genes was significantly downregulated (5 of 15 genes).

The IFN genes were normally expressed in the monocytes of the BD patients.

The expression of the GRα and HGF gene was significantly upregulated in the monocytes of BD patients as compared to those of HC (*p* = 0.039 and *p* = 0.004, respectively) (Table 2).

Table 3 shows that the expression level of HGF in monocytes correlated significantly positive with the expression of the activating GRα and with the IFN cluster, but negative with the expression levels of the cluster 1 and 2 genes: the higher the HGF expression the higher the GRα expression and the IFN expression, but the lower the expression of IL1, CCL20 and IL6, and NAB2, CCL2, and CCL7.

**Table 3:** **Correlations of hair cortisol, glucocorticoid receptor (GR) α and GRβ receptor gene expression, hepatocyte growth factor (HGF) gene expression, and cluster 1-, 2- and interferon-related cluster scores**.

	HGF	Hair cortisol	GRα	IL1B	CCL20	IL6	CCL7	NAB2	CCL2	GRβ	IFI44	IFIT3
HGF		0.21	0.34	−0.42	−0.38	−0.50	−0.43	−0.53	−0.35	−0.18	0.18	0.41
Hair cortisol	0.21		0.12	−0.12	−0.06	−0.05	0.00	−0.14	−0.02	0.13	0.14	0.19
GRα	0.34	0.12		−0.39	−0.24	−0.30	−0.10	−0.20	−0.15	−0.04	0.12	0.25
GRβ	−0.18	0.13	−0.04	0.35	0.38	0.40	0.34	0.38	0.33		0.11	−0.07

*Correlations significant at the p = 0.05 level are colored*.

Table 3 also shows that the gene expression of the blocking GRβ expression in the monocytes of the patients correlated positive to the cluster 1 gene expression, an observation reported before (7, 31).

The disease characteristics as given in Table 1, including the mood state, the medication, and the presence of the MetS, did not correlate to the various gene expression levels in the monocytes of the BD patients (data not shown).

In the correlations between hair cortisol and other parameters, we used an analogous approach. The hair cortisol levels did not significantly correlate to any of the above described immune parameters (Table 3). We did, however, confirm the correlation of the hair cortisol with negative life events in the previous months to bloodletting as reported earlier by Staufenbiel et al. (17).

### Leukocyte Subset Determinations

For leukocyte subset determination, inter-assay differences performed on PBMC in the various cohorts of MOODINFLAME and PSYCHAID and obtained at different sites and collection times appeared to be negligible, therefore a total number of 519 HC with PBMC data were available for evaluation against the BiSS cases; however, only HC with complete information on gender, age, and BMI (*n* = 272) were included for comparison with the BiSS cases. Out of these 272 HC, a selection was made to match for age (maximum 3-year difference), gender, and BMI (maximum three BMI points difference) with the BiSS patient group; this resulted in 72 pairs (BD cases–HC pair) for leukocyte subset determination. The mean age of these pairs was 52 years (range 20–67), percentage females 63, and mean BMI 25.3 (SD 3.2) not statistically different from the entire group of BiSS BD patients (Table 1).

Table 4 shows that there was a reduction of the percentage of lymphocytes in the BD patients. The percentage of monocytes was raised. Since many of the patients were on lithium, we carried out a separate comparison between lithium-treated and non-lithium-treated BD patients. This comparison did not show an effect of lithium treatment (see Table 4). Correlations with the usage of other drugs were also not found (data not shown).

**Table 4:** **Peripheral blood mononuclear cells (PBMCs) in bipolar disorder (BD) patients compared to matched healthy controls (HCs)**.

	All HC (*n* = 72)		All BD patients (*n* = 72)			HC no lithium^b^ (*n* = 16)		BD no lithium (*n* = 16)			HC lithium^d^ (*n* = 56)		BD lithium (*n* = 56)		
	Mean	SD	Mean	SD	*p*^a^	Mean	SD	Mean	SD	*p*^c^	Mean	SD	Mean	SD	*p*
Lymphocytes	76.85	0.88	72.54	0.94	**<0.001**	79.81	1.33	76.28	1.72	0.088	76.00	1.06	71.47	1.08	**0.003**
CD19^+^ B cells	7.32	0.28	7.56	0.30	0.56	6.65	0.50	7.38	0.60	0.32	7.52	0.32	7.61	0.36	0.84
CD56^+^ NK cells	9.11	0.58	10.21	0.64	0.19	10.75	1.30	10.64	1.59	0.95	8.62	0.65	10.10	0.71	0.12
CD14^+^ monocytes	17.63	0.74	21.01	0.70	**0.001**	14.23	1.19	18.62	1.54	0.020	18.54	0.85	21.72	0.80	**0.007**
CD3^+^ T cells	57.66	0.99	53.31	1.17	**0.005**	59.98	1.80	56.53	2.67	0.25	56.99	1.19	52.38	1.32	**0.009**
CD3^+^CD8^+^ cytotoxic T cells	16.31	0.69	14.65	0.67	0.083	18.50	1.72	16.16	1.81	0.32	15.68	0.76	14.22	0.72	0.11
CD3^+^CD4^+^ T-helper cells	38.42	0.90	36.18	1.11	0.11	38.74	2.33	37.53	2.37	0.69	38.33	0.98	35.79	1.29	0.15
CD3^+^CD4^+^CD25^hi^FoxP3^+^ Treg	1.56	0.07	1.46	0.05	0.21	1.51	0.18	1.60	0.13	0.65	1.58	0.07	1.41	0.06	0.076
CD3^+^CD4^+^IFN-γ^+^ T-helper 1 cells	4.36	0.26	4.43	0.29	0.73	4.78	0.61	5.27	0.89	0.80	4.23	0.29	4.19	0.29	0.80
CD3^+^CD4^+^IL4^+^ T-helper 2 cells	0.43	0.03	0.53	0.04	**0.037**	0.53	0.10	0.41	0.07	0.26	0.40	0.03	0.56	0.04	**0.002**
CD3^+^CD4^+^IL17A^+^ T-helper 17 cells	0.25	0.02	0.33	0.03	**0.003**	0.25	0.03	0.30	0.03	0.18	0.25	0.02	0.34	0.04	**0.009**

*Percentages of indicated subpopulations are given per PBMC (mononuclear white cells). The *p* values in comparison of the lithium and not-lithium using BD patients versus their respective controls are given below*.

*Values marked in bold indicate numbers that are significant on the 95% confidence limit*.

*p^a^: all BD patients versus all matched HCs*.

*HC no lithium^b^: matched HCs corresponding to BD patients with no lithium usage*.

*p^c^: BD patients without lithium usage versus corresponding matched HC*.

*HC lithium^d^: matched HCs corresponding to BD patients with lithium usage*.

With regard to the subpopulations of lymphocytes, Table 4 shows that it was particularly the CD3^+^ (pan) T cells, which were decreased and within this population both the CD3^+^CD8^+^ cytotoxic T cells and the CD3^+^CD4^+^ T-helper cells. Within the population of CD3^+^CD4^+^ T-helper cells a shift had taken place, there was a significant increase in both the percentages of Th2 and Th17 cells, while the Treg cells had decreased. These abnormalities were also present in patients not on lithium, except for the Th2 increase; this was only seen in the lithium treated BD patients.

B cell, NK cell, and Th1 cell levels in the BD patients were not different from the HCs.

In the correlations between hair cortisol and the leukocyte subsets, we again used an analogous approach. Correlations between hair cortisol and any of the leukocyte subsets could not be detected. This also applied for correlations between the leukocyte subsets and clinical characteristics of the patients, including the mood state and the presence of the MetS (data not shown).

## Discussion

This study shows that the pro-inflammatory gene cluster 1 expression was downregulated in the monocytes of the BD patients of the here studied BiSS cohort, indicating a reduced inflammatory state of the circulating monocytes as compared to that of HCs.

This reduced inflammatory state of the circulating monocytes of the BiSS patients is in contrast to the pro-inflammatory state observed in previously studied cohorts of BD patients while in all cohorts the same collection and laboratory determination techniques were used. In the BD patients of the D-SFBN cohort, an increased inflammatory gene expression was found that correlated to the activity of disease, particularly to manic symptoms (2, 3, 6). The patients in the D-SFBN cohort were indeed special in that a relative high percentage had active severe disease (38%). In the MOODINFLAME Leuven–Groningen cohort, the raised pro-inflammatory gene expression in monocytes was again only found in patients with active mania or depression, these patients constituted around 10% of the cohort (4). Our data thus support a concept that in BD patients over expression of monocyte inflammatory genes can only be found during active episodes of the disease, in particular during mania. The BiSS cohort only had six patients with severe depression.

Our observations on monocytes are by and large in accord with serum cytokine/inflammatory factor studies that also found that BD patients with an active episode and particularly those who have or develop manic symptoms have an increased inflammatory activity, especially high CRP, compared with individuals who do not develop manic symptoms (34).

However, before to conclude that the pro-inflammatory state in BD is only linked to (manic) mood derailment in BD, it is relevant that in the studies on the DBO children over expression of monocyte inflammatory genes was also found in adolescents at 16 years of age, but in this case irrespective of present or later mood symptoms (5). Five years later, in early adulthood (at 21 years of age), the pattern of monocyte gene expression had changed in the DBO children to a reduced expression of cluster 1 genes, but still increased cluster 2 gene upregulation. At 28 years, monocyte gene expression had virtually normalized. Neither increases nor decreases in monocyte inflammatory gene expression were linked to mood symptomatology in the DBO children in the various stages of investigation.

Collectively, these findings on the inflammatory state of monocytes point in the direction of dynamic changes of inflammatory gene expression in monocytes over time in subjects at risk to develop BD and in BD patients. Apparently the inflammatory set point of monocytes is dependent on the (environmental or hormonal) state of the organism, an observation that is supported by twin studies in BD, showing that common endogenous or environmental factors play a prime role in the pro-inflammatory set point of the monocytes (35). In sum, only episodes of pro-inflammatory activation occur in circulating monocytes of individuals at risk to develop BD and in BD patients, and these episodes are probably environmentally and endogenously precipitated and only in part linked to the presence and activity of (manic) mood symptoms.

The present study also shows that the gene expression of the vascular repair factor HGF was significantly raised in the monocytes of BD patients of the BiSS cohort as compared to HCs. We previously found a similar gene expression profile, i.e., a reduced pro-inflammatory cluster 1 and raised HGF expression, in the circulating monocytes of 64 T2D patients (23, 36). These T2D patients were on average 62 years old.

We took the observation in the studies of Baldóon Rojas et al. of a high HGF expression and a low expression of inflammatory signs in the circulating monocytes of T2D patients as indicating that many of the circulating monocytes in the T2D cases were differentiating into CACs and involved in the repair of endothelium damaged by the diabetic process. CACs are bone marrow-derived monocytes involved in vascular regeneration and angiogenesis, are anti-inflammatory, and exert their repair function by secreting vascular growth factors, such as HGF, which is considered as a marker of CACs (25). Healthy angiogenesis is crucial for vascular regeneration (24).

We therefore like to hypothesize that the elevated gene expression of HGF in the anti-inflammatory monocytes of the patients of the BiSS cohort probably reflects that a high percentage of the monocytes had developed into vessel-repair-supporting CACs to counteract a putative endothelial damage in the BD patients of the BiSS cohort. Indeed, the MetS and dyslipidemia were prevalent in the BD patients of the BiSS cohort (18), yet there were no correlations between the HGF expression and the MetS. Therefore, formal studies in older BD patients focusing on monocyte-derived CAC development in relation to parameters of vascular damage need to be performed to verify or refute the here proposed idea of the development of anti-inflammatory CACs as overruling mechanism preventing a pro-inflammatory state of monocytes in BD patients. Also endothelial damage in atherosclerosis is considered a pro-inflammatory environment (37), and it is therefore at first sight puzzling that the CACs are anti-inflammatory. However, there is literature that it is particularly in situations of chronic mild hyperglycemia (38) and in situations of unstable plaque formation (39) that CACs and circulating monocytes become pro-inflammatory and dysfunctional. Therefore, special care should be taken in follow-up studies whether such conditions exist in bipolar patients next to a putative heightened endothelial damage.

The GRα was also higher expressed in the monocytes of the BD patients of the BiSS cohort as compared to those of HCs, and the expression of the GRα expression correlated to the expression of HGF. Since the GRα is the activating receptor signaling downregulation of pro-inflammatory molecules during glucocorticoid exposure, an upregulation of the GRα suggests a higher sensitivity for glucocorticoids to downregulate the inflammatory state of monocytes.

These data suggest that in principle a raised cortisol level might be instrumental in downregulating the inflammatory state of the circulating monocytes in BD patients. Such a raised cortisol level was reported in BiSS BD patients who experienced recent negative life events (17). However, although we were able to verify the previous correlation between high hair cortisol levels and negative life events, we were unable to find in this study a correlation between both high hair cortisol levels/negative life events on the one hand and a reduced inflammatory state of the circulating monocytes of the BiSS cohort on the other hand.

We therefore assume that not negative life events and a higher HPA-axis activity, but the earlier mentioned putative endothelial damage and consequent transition of the circulating monocytes into anti-inflammatory vessel repair cells CACs plays a overruling and prime role in the here found characteristic profile (high HGF, reduced pro-inflammatory compounds) of the circulating monocytes in the BD patients of the BiSS cohort. But as stated before, this needs formal verification, also with regard to an enhanced monocyte sensitivity to dexamethasone in functional *in vitro* exposure tests.

With regard to the T cell findings in the BiSS cohort the following remarks. Snijders et al. (12) took the monocyte data on the DBO adolescents of Mesman et al. (5) further and showed that the DBO 16-year-old adolescents had deficits in the number of circulating lymphocytes, particularly of total CD3^+^ T cells and within this population particularly in the Treg cell subset. Interestingly, she found that the lower the level of the anti-inflammatory Treg cells in the DBO adolescents, the higher the monocyte inflammatory gene activation. These data corroborate earlier findings that Treg cells are capable of downregulating monocyte inflammatory activation and that in particular a defective Treg cell system might form the underlying condition allowing endogenous or environmental factors to induce an early raised pro-inflammatory state of monocytes in the 16-year-old DBO subjects at risk to develop BD.

During further aging of the DBO children, the aberrant T cell state showed a dynamic course over time. During young adulthood (mean age 21 years), the partial T cell deficit, i.e., the reduction in the total number of CD3^+^ T cell was still detectable, but not of the Treg cell subset anymore, but of the pro-inflammatory Th1 and Th17 cell subsets, suggesting a phase of relative anti-inflammation at young adulthood, but in the presence of deficits of total T cells. Also the upregulation of cluster 1 inflammatory genes in monocytes had disappeared and even a downregulation of such genes as compared to HC monocytes was found (see before), enforcing the idea that young adulthood at 21 years of age was a phase of relative anti-inflammation.

In late adulthood (mean age 28 years), the various subsets of T cells (Th1, Th2, Th17, and Treg cells) and the abnormal monocyte gene expression had largely normalized as compared to the controls, only the deficit of lymphocytes and total CD3^+^ T cells could still be detected.

**Figure 1:**
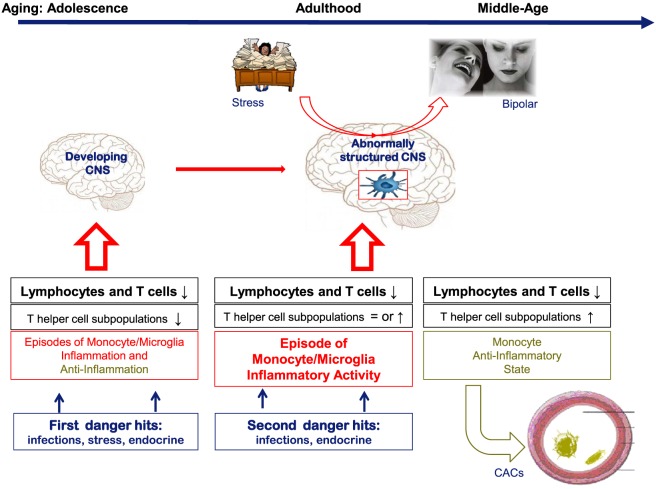
**A hypothetical cartoon on the natural history of the lymphocyte and monocyte changes in bipolar subjects over time and their consequences for brain function and support function of blood vessels**. In the cartoon, it is assumed that in healthy conditions both T cells and monocytes/microglia are essential for proper brain development and function. When there is a deficit in T cells or changing inflammatory states as in bipolar disorder (BD) and the pre-stages of BD, we assume that brain development and function are abnormal leading to a vulnerability for mood derailment.

Collectively, these DBO data show that T cell deficits deregulate the inflammatory response in adolescence and young adulthood in individuals at risk for BD, and we assume that the immune disequilibria represent vulnerability factors for later BD development by influencing morphological brain and hippocampus development during adolescence and early adulthood, since T cells and non-inflammatory microglia are essential support cells for proper brain development (see Figure 1).

Although the T cell and monocyte inflammatory disequilibria were more or less normalized in adulthood in the DBO subjects, a partial T cell defect (a deficit in lymphocyte and total T cell numbers) still existed. This report on the BiSS BD patients with a mean age of 52 years still finds the apparently over time consistent partial T cell deficit in showing reduced levels of lymphocytes and total CD3^+^ T cells also in the relatively old BiSS cohort, while the restoration within the CD4^+^ T-helper cells subset had further continued into an overshoot, showing significantly raised levels of Th2 and Th17 subpopulations, while Treg cells were reduced.

A composite of the dynamics over time of T cells and monocytes as found in the various cohorts studied by us is shown in Figure 1. In this figure, it is also hypothesized that particularly in adolescence and early adulthood changes in brain structure and physiology are induced by the T cell deficits and inflammatory influences. In later phases of life, partial corrections of the immune aberrancies occur, and only during an active episode signs of a peripheral monocyte inflammatory state can be found.

### Limitations of This Study

Data on the leukocyte subpopulations could only be given as percentages of the total number of leukocytes or lymphocytes. Absolute counts of the same leukocyte populations were not performed. Future studies should focus on more exact and extensive measurements of the actual circulating numbers of discriminating leukocyte populations.

Vasculature related factors have not been measured in the BiSS cohort, such as endothelin and the carotid intima thickness, which would have given a better impression of the state of the endothelium. Future studies should involve patients with a better metabolic characterization and characterization of the state of their vasculature.

Another limitation is that the exclusion criteria for patients and controls were not exactly the same. Patients of the BiSS cohort were not excluded if known with an endocrine or immune disease. However, endocrine diseases were recorded in the BiSS group (seven patients had thyroid disease and three diabetes), and of note the presence of endocrine disease did not correlate with immune outcomes (data not shown). Yet future studies should try and match as much as possible for the various conditions having an effect on the immune state of both patients and controls (age, gender, BMI, comorbidities, smoking, stress, etc.).

## Conclusion

Despite the limitations, we are confident that this study shows that lymphopenia, and in particular a reduced percentage of T cells, is a characteristic of relatively old BD patients. Since lymphopenia and a reduced percentage of T cells are phenomena also found in children at risk for BD and younger BD patients, a deficit of T cells might be a trait phenomenon of BD.

The monocyte gene expression profile of the relatively old BD patients showed a downregulation of inflammatory genes and an upregulation of HGF, suggesting CAC activity of the cells. This profile is different from the pro-inflammatory profile found in 16-year-old offspring of a bipolar parent and in BD patients with active disease. Therefore, the changes in monocyte profiles suggest dynamic changes over time in the inflammatory state of monocytes of individuals at risk for manic-depressive episodes. We therefore assume that the monocyte inflammatory set point is a state rather that a trait phenomenon of BD.

## Author Contributions

All the authors collected part of the study cohort, evaluated the data, particularly the hair cortisol and metabolic parameters, and assisted in writing of the manuscript.

## Conflict of Interest Statement

The authors declare that the research was conducted in the absence of any commercial or financial relationships that could be construed as a potential conflict of interest.

## References

[1] Modabbernia A Taslimi S Brietzke E Ashrafi M. Cytokine alterations in bipolar disorder: a meta-analysis of 30 studies. Biol Psychiatry (2013) 74(1):15–25. 10.1016/j.biopsych.2013.01.007 23419545

[2] Padmos RC Hillegers MH Knijff EM Vonk R Bouvy A Staal FJ A discriminating messenger RNA signature for bipolar disorder formed by an aberrant expression of inflammatory genes in monocytes. Arch Gen Psychiatry (2008) 65(4):395–407. 10.1001/archpsyc.65.4.395 18391128

[3] Drexhage RC van der Heul-Nieuwenhuijsen L Padmos RC van Beveren N Cohen D Versnel MA Inflammatory gene expression in monocytes of patients with schizophrenia: overlap and difference with bipolar disorder. A study in naturalistically treated patients. Int J Neuropsychopharmacol (2010) 13(10):1369–81. 10.1017/S1461145710000799 20633309

[4] Becking K Haarman BC van der Lek RF Grosse L Nolen WA Claes S Inflammatory monocyte gene expression: trait or state marker in bipolar disorder? Int J Bipolar Disord (2015) 3(1):20. 10.1186/s40345-015-0037-x 26381439 PMC4574035

[5] Mesman E Hillegers MH Ambree O Arolt V Nolen WA Drexhage HA. Monocyte activation, brain-derived neurotrophic factor (BDNF), and S100B in bipolar offspring: a follow-up study from adolescence into adulthood. Bipolar Disord (2015) 17(1):39–49. 10.1111/bdi.12231 25039314

[6] Haarman BC Riemersma-Van der Lek RF Burger H Netkova M Drexhage RC Bootsman F Relationship between clinical features and inflammation-related monocyte gene expression in bipolar disorder – towards a better understanding of psychoimmunological interactions. Bipolar Disord (2014) 16(2):137–50. 10.1111/bdi.12142 24286609

[7] Bergink V Burgerhout KM Weigelt K Pop VJ de Wit H Drexhage RC Immune system dysregulation in first-onset postpartum psychosis. Biol Psychiatry (2013) 73(10):1000–7. 10.1016/j.biopsych.2012.11.006 23270599

[8] Weigelt K Bergink V Burgerhout KM Pescatori M Wijkhuijs A Drexhage HA Down-regulation of inflammation-protective microRNAs 146a and 212 in monocytes of patients with postpartum psychosis. Brain Behav Immun (2013) 29:147–55. 10.1016/j.bbi.2012.12.018 23295264

[9] Gardiner E Beveridge NJ Wu JQ Carr V Scott RJ Tooney PA Imprinted DLK1-DIO3 region of 14q32 defines a schizophrenia-associated miRNA signature in peripheral blood mononuclear cells. Mol Psychiatry (2012) 17(8):827–40. 10.1038/mp.2011.78 21727898 PMC3404364

[10] Serafini G Pompili M Hansen KF Obrietan K Dwivedi Y Shomron N The involvement of microRNAs in major depression, suicidal behavior, and related disorders: a focus on miR-185 and miR-491-3p. Cell Mol Neurobiol (2014) 34(1):17–30. 10.1007/s10571-013-9997-5 24213247 PMC11488878

[11] Drexhage RC Hoogenboezem TH Versnel MA Berghout A Nolen WA Drexhage HA. The activation of monocyte and T cell networks in patients with bipolar disorder. Brain Behav Immun (2011) 25(6):1206–13. 10.1016/j.bbi.2011.03.013 21443944

[12] Snijders G Schiweck C Mesman E Grosse L De Wit H Nolen WA A dynamic course of T cell defects in individuals at risk for mood disorders. Brain Behav Immun (2016) 58:11–7. 10.1016/j.bbi.2016.05.007 27181178

[13] Koenders MA Giltay EJ Spijker AT Hoencamp E Spinhoven P Elzinga BM. Stressful life events in bipolar I and II disorder: cause or consequence of mood symptoms? J Affect Disord (2014) 161:55–64. 10.1016/j.jad.2014.02.036 24751308

[14] Spijker AT van Rossum EF Hoencamp E DeRijk RH Haffmans J Blom M Functional polymorphism of the glucocorticoid receptor gene associates with mania and hypomania in bipolar disorder. Bipolar Disord (2009) 11(1):95–101. 10.1111/j.1399-5618.2008.00647.x 19133972

[15] Spijker AT Giltay EJ van Rossum EF Manenschijn L DeRijk RH Haffmans J Glucocorticoid and mineralocorticoid receptor polymorphisms and clinical characteristics in bipolar disorder patients. Psychoneuroendocrinology (2011) 36(10):1460–9. 10.1016/j.psyneuen.2011.03.020 21531081

[16] Manenschijn L Spijker AT Koper JW Jetten AM Giltay EJ Haffmans J Long-term cortisol in bipolar disorder: associations with age of onset and psychiatric co-morbidity. Psychoneuroendocrinology (2012) 37(12):1960–8. 10.1016/j.psyneuen.2012.04.010 22634056

[17] Staufenbiel SM Koenders MA Giltay EJ Elzinga BM Manenschijn L Hoencamp E Recent negative life events increase hair cortisol concentrations in patients with bipolar disorder. Stress (2014) 17(6):451–9. 10.3109/10253890.2014.968549 25243794

[18] Silarova B Giltay EJ Van Reedt Dortland A Van Rossum EF Hoencamp E Penninx BW Metabolic syndrome in patients with bipolar disorder: comparison with major depressive disorder and non-psychiatric controls. J Psychosom Res (2015) 78(4):391–8. 10.1016/j.jpsychores.2015.02.010 25742722

[19] Foguet-Boreu Q Fernandez San Martin MI Flores Mateo G Zabaleta Del Olmo E Ayerbe García-Morzon L Perez-Piñar López M Cardiovascular risk assessment in patients with a severe mental illness: a systematic review and meta-analysis. BMC Psychiatry (2016) 16:141. 10.1186/s12888-016-0833-6 27176477 PMC4866037

[20] Knijff EM Breunis MN van Geest MC Kupka RW Ruwhof C de Wit HJ A relative resistance of T cells to dexamethasone in bipolar disorder. Bipolar Disord (2006) 8(6):740–50. 10.1111/j.1399-5618.2006.00359.x 17156159

[21] Bei E Salpeas V Pappa D Anagnostara C Alevizos V Moutsatsou P. Phosphorylation status of glucocorticoid receptor, heat shock protein 70, cytochrome c and Bax in lymphocytes of euthymic, depressed and manic bipolar patients. Psychoneuroendocrinology (2009) 34(8):1162–75. 10.1016/j.psyneuen.2009.03.002 19359101

[22] Barbosa IG Machado-Vieira R Soares JC Teixeira AL. The immunology of bipolar disorder. Neuroimmunomodulation (2014) 21(2–3):117–22. 10.1159/000356539 24557044 PMC4041530

[23] Baldeón Rojas L Weigelt K de Wit H Ozcan B van Oudenaren A Sempértegui F Study on inflammation-related genes and microRNAs, with special emphasis on the vascular repair factor HGF and miR-574-3p, in monocytes and serum of patients with T2D. Diabetol Metab Syndr (2016) 8:6. 10.1186/s13098-015-0113-5 26779287 PMC4714426

[24] Taniyama Y Morishita R Aoki M Nakagami H Yamamoto K Yamazaki K Therapeutic angiogenesis induced by human hepatocyte growth factor gene in rat and rabbit hindlimb ischemia models: preclinical study for treatment of peripheral arterial disease. Gene Ther (2001) 8(3):181–9. 10.1038/sj.gt.3301379 11313789

[25] Bouchentouf M Paradis P Forner KA Cuerquis J Boivin MN Zheng J Monocyte derivatives promote angiogenesis and myocyte survival in a model of myocardial infarction. Cell Transplant (2010) 19(4):369–86. 10.3727/096368909X484266 20021736

[26] Lecrubier Y Sheehan DV Weiller E Amorim P Bonora I Harnett Sheehan K The Mini International Neuropsychiatric Interview (MINI). A short diagnostic structured interview: reliability and validity according to the CIDI. Eur Psychiatry (1997) 12:224–31. 10.1016/S0924-9338(97)83296-8

[27] Rush AJ Trivedi MH Ibrahim HM Carmody TJ Arnow B Klein DN The 16-item quick inventory of depressive symptomatology (QIDS), clinician rating (QIDS-C), and self-report (QIDS-SR): a psychometric evaluation in patients with chronic major depression. Biol Psychiatry (2003) 54(5):573–83. 10.1016/S0006-3223(02)01866-8 12946886

[28] Young RC Biggs JT Ziegler VE Meyer DA. A rating scale for mania: reliability, validity and sensitivity. Br J Psychiatry (1978) 133:429–35. 10.1192/bjp.133.5.429 728692

[29] Expert Panel on Detection, Evaluation, and Treatment of High Blood Cholesterol in Adults. Executive summary of the third report of the national cholesterol education program (NCEP) expert panel on detection, evaluation, and treatment of high blood cholesterol in adults (adult treatment panel III). JAMA (2001) 285(19):2486–97. 10.1001/jama.285.19.2486 11368702

[30] Manenschijn L Koper JW van den Akker EL de Heide LJ Geerdink EA de Jong FH A novel tool in the diagnosis and follow-up of (cyclic) Cushing’s syndrome: measurement of long-term cortisol in scalp hair. J Clin Endocrinol Metab (2012) 97(10):E1836–43. 10.1210/jc.2012-1852 22844063

[31] Carvalho LA Bergink V Sumaski L Wijkhuijs J Hoogendijk WJ Birkenhager TK Inflammatory activation is associated with a reduced glucocorticoid receptor alpha/beta expression ratio in monocytes of inpatients with melancholic major depressive disorder. Transl Psychiatry (2014) 4:e344. 10.1038/tp.2013.118 24424390 PMC3905228

[32] Applied Biosystems. User Bulletin #2, Applied Biosystems PRISM 7700 Sequence Detection System: Relative Quantitation of Gene Expression. (1997). Available from: http://www3.appliedbiosystems.com/cms/groups/mcb_support/documents/generaldocuments/cms_040980.pdf

[33] Brkic Z Maria NI van Helden-Meeuwsen CG van de Merwe JP van Daele PL Dalm VA Prevalence of interferon type I signature in CD14 monocytes of patients with Sjogren’s syndrome and association with disease activity and BAFF gene expression. Ann Rheum Dis (2013) 72(5):728–35. 10.1136/annrheumdis-2012-201381 22736090 PMC3618683

[34] Becking K Boschloo L Vogelzangs N Haarman BC Riemersma-van der Lek R Penninx BW The association between immune activation and manic symptoms in patients with a depressive disorder. Transl Psychiatry (2013) 3:e314. 10.1038/tp.2013.87 24150223 PMC3818012

[35] Padmos RC Van Baal GC Vonk R Wijkhuijs AJ Kahn RS Nolen WA Genetic and environmental influences on pro-inflammatory monocytes in bipolar disorder: a twin study. Arch Gen Psychiatry (2009) 66(9):957–65. 10.1001/archgenpsychiatry.2009.116 19736352

[36] Baldeón RL Weigelt K de Wit H Ozcan B van Oudenaren A Sempértegui F Type 2 diabetes monocyte microRNA and mRNA expression: dyslipidemia associates with increased differentiation-related genes but not inflammatory activation. PLoS One (2015) 10(6):e0129421. 10.1371/journal.pone.0129421 26083362 PMC4471054

[37] Hansson GK Robertson AK Söderberg-Nauclér C. Inflammation and atherosclerosis. Annu Rev Pathol (2006) 1:297–329. 10.1146/annurev.pathol.1.110304.100100 18039117

[38] Loomans CJ van Haperen R Duijs JM Verseyden C de Crom R Leenen PJ Differentiation of bone marrow-derived endothelial progenitor cells is shifted into a proinflammatory phenotype by hyperglycemia. Mol Med (2009) 15(5–6):152–9. 10.2119/molmed.2009.00032 19295918 PMC2656993

[39] Falk E Nakano M Bentzon JF Finn AV Virmani R. Update on acute coronary syndromes: the pathologists’ view. Eur Heart J (2013) 34(10):719–28. 10.1093/eurheartj/ehs411 23242196

