# Supplementary material for: T Cell Deficits and Overexpression of Hepatocyte Growth Factor in Anti-inflammatory Circulating Monocytes of Middle-Aged Patients with Bipolar Disorder Characterized by a High Prevalence of the Metabolic Syndrome
Source: Front Psychiatry. 2017 Mar 20;8:34. doi: 10.3389/fpsyt.2017.00034 (PMC5357747; doi:10.3389/fpsyt.2017.00034)
Supplement: Supplementary file 1 [file Data_Sheet_1.docx]

| 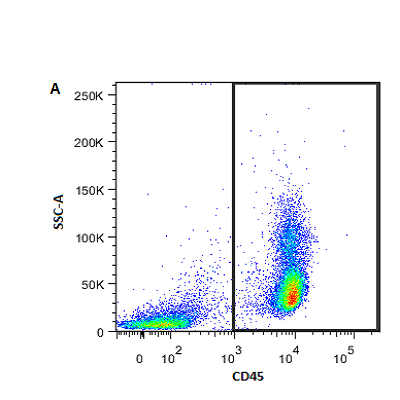 | 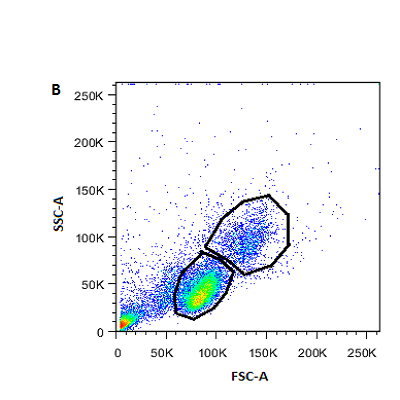 | 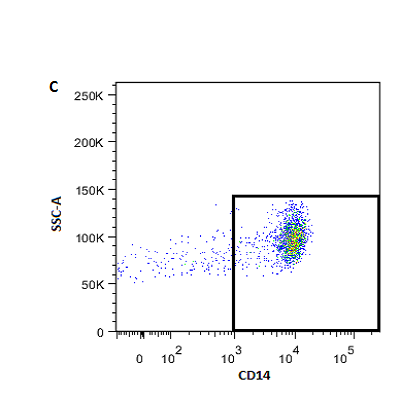 |
| --- | --- | --- |
| 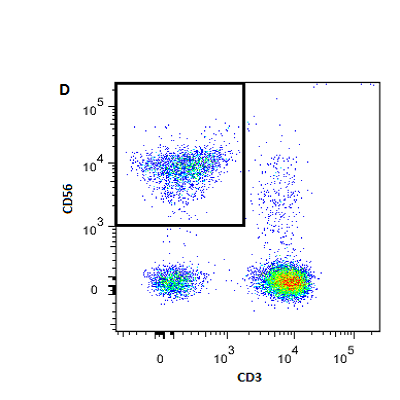 | 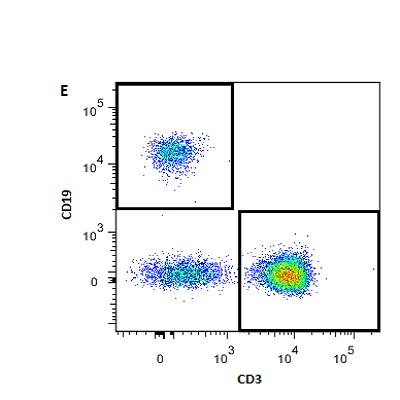 | 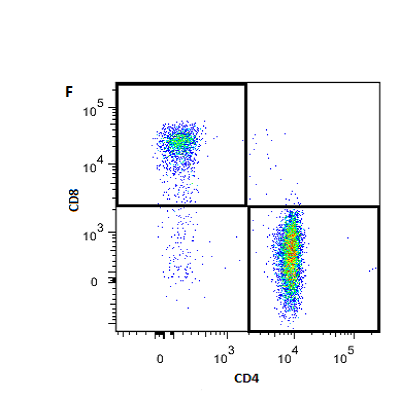 |
| 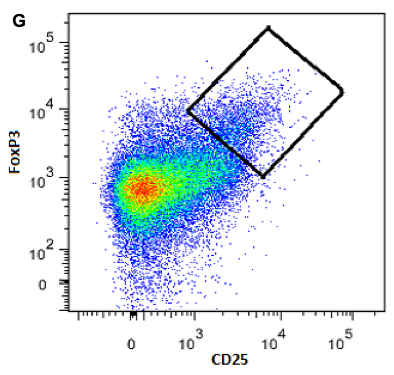 | 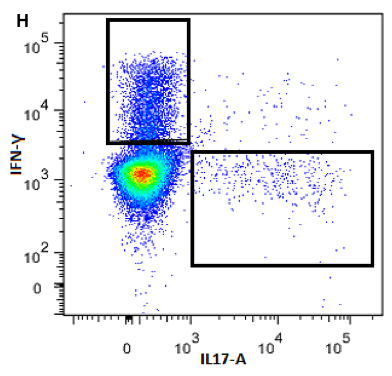 | 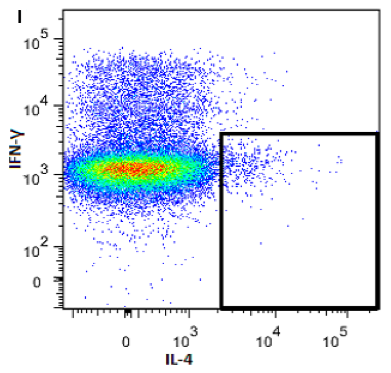 |

**Supplementary figure 1 – gating strategies for determination of T-cells and T-cell subsets**

Definition of PBMC subsets and gating strategy. **(A)** Selection of leukocytes. Gating was performed on CD45+ events, excluding platelets, erythrocytes and cell debris. **(B)** Selection of total lymphocytes (lower gate) and monocytes (upper gate) based of forward scatter (FSC-A) and side scatter (SSC-A). **(C)** Selection of CD14+ monocytes within the monocyte gate. **(D)** Selection of CD3-CD56+ natural killer cells within the lymphocyte gate. **(E)** Selection of T (CD3+ events, lower right gate) and B cells (CD19+ events, upper left gate) within the lymphocyte gate. **(F)** Selection of CD3+CD4+ T-helper cells (lower right gate) and CD3+CD8+ cytotoxic T cells (upper left gate). **(G)** Selection of CD3^+^CD4^+^CD25^hi^FoxP3^+^ T-regulatory cells within the T cell gate. **(H)** Selection of CD3^+^CD4^+^IFN-γ^+^ Th1 cells (upper left gate) and CD3^+^CD4^+^IL17A^+^  Th17 cells (lower right gate) within the helper T cell gate. **(I)** Selection of CD3^+^CD4^+^IL4^+^ Th2 cells within the helper T cell gate.

| **Supplementary table 1.** List of genes included in qPCR for determination of monocyte gene expression.  These genes have been found abnormally expressed in the monocytes in previous studies on various mood disorder patient groups (see text), some consistently, other occasionally. We therefore used the genes in this study again. | | |
| --- | --- | --- |
|  |  |  |
| **Gene** | **TaqMan assay ID** | **Corresponding protein** |
| ADM | Hs00181605_m1 | Adrenomedulin |
| ATF3 | Hs00231069_m1 | Cyclic AMP-dependent transcription factor 3 |
| BCL2A1 | Hs00187845_m1 | B cell lymphoma 2-related protein A1 |
| BTG3 | Hs00199064_m1 | BTG family, member 3 |
| CCL2 | Hs00234140_m1 | C-C chemokine ligand 2 |
| CCL20 | Hs00355476_m1 | C-C chemokine ligand 20 |
| CCL7 | Hs00171147_m1 | C-C chemokine ligand 7 |
| CD9 | Hs00233521_m1 | Cluster of differentiation |
| CDC42 | Hs00741586_mH | Cell division control protein 42 homolog |
| CXCL2 | Hs00236966_m1 | C-X-C chemokine ligand 2 |
| DHRS3 | Hs00191073_m1 | Short-chain dehydrogenase/reductase 3 |
| DUSP2 | Hs00358879_m1 | Dual specificity protein phosphatase 2 |
| EGR3 | Hs00231780_m1 | Early Growth Response 3 |
| EMP1 | Hs00608055_m1 | Epithelial Membrane Protein 1 |
| EREG | Hs00914313_m1 | Epiregulin |
| HGF | Hs00300159_m1 | Hepatocyte growth factor |
| HSPA1A/B | Hs00271229_s1 | Heat shock 70kDa protein 1 |
| IFI44 | Hs00197427_m1 | Interferon Induced Protein 44 |
| IFIT3 | Hs00382744_m1 | Interferon Induced Protein With Tetratricopeptide Repeats 3 |
| IL1A | Hs00174092_m1 | Interleukin 1α |
| IL1B | Hs00174097_m1 | Interleukin 1β |
| IL1R1 | Hs00168392_m1 | Interleukin 1 receptor, type 1 |
| IL6 | Hs00174131_m1 | Interleukin 6 |
| IRAK2 | Hs00176394_m1 | Interkeukin 1 receptor-associated kinase-like 2 |
| MAFF | Hs00202412_m1 | Musculoaponeurotic fibrosarcoma oncogene homolog F |
| MAPK6 | Hs00833126_g1 | Mitogen-activated protein kinase 6 |
| MXD1 | Hs00231137_m1 | MAD protein |
| NAB2 | Hs00195573_m1 | Nerve growth factor-induced protein A-binding protein 2 |
| NR3C1α | Hs00353740_m1 | Nuclear Receptor Subfamily 3 Group C Member 1 α (glucocorticoid receptor α) |
| NR3C1β | Hs00354508_m1 | Nuclear Receptor Subfamily 3 Group C Member 1 β (glucocorticoid receptor β) |
| PDE4B | Hs00387320_m1 | cAMP-specific 3',5'-cyclic phosphodiesterase 4B |
| PTGS2 | Hs00153133_m1 | Prostoglandin G/H synthase (cyclooxygenase) |
| PTPN7 | Hs00160732_m1 | Protein tyrosine phosphatase, non-receptor type 7 |
| PTX3 | Hs00173615_m1 | Pentraxin-related protein 3 |
| SERPINB2 | Hs00234032_m1 | Plasminogen activator inhibitor-2 |
| STX1A | Hs00270282_m1 | Syntaxin-1A |
| THBD | Hs00264920_s1 | Thrombomodulin |
| TNF | Hs00174128_m1 | Tumor nectoris factor |
| TNFAIP3 | Hs00234712_m1 | Tumor nectoris factor, alpha-induced protein 3 |

**Supplementary table 2.** **Principle component analysis for grouping of monocytes.**

Shown are the pattern matrices, numbers are the correlations between the genes and components. Three fixed factors were extracted for both the HC and BD analyses, using oblimin rotation with a maximum of 25 iterations and listwise deletion of participants without complete data for all included genes.

The three found clusters in principle component analysis were largely similar to the clusters found in our previous studies on mood disorder patients (see text), though some of the lower ranking genes of cluster 1 (like HSPA1A/B) and of cluster 2 (like CD9) in the previous studies did not belong to these clusters in the present study anymore. We assume that in this study the power is insufficient to assign these genes to the molecular pathways due to their loose connection to the pathway.

|  | **Component** | | |
| --- | --- | --- | --- |
|  | **1** | **2** | **3** |
| **IL1B** | .978 | -.105 | -.125 |
| **CCL20** | .937 | -.048 | -.049 |
| **IL6** | .907 | -.043 | -.125 |
| **TNFAIP3** | .870 | -.282 | .136 |
| **CXCL2** | .739 | .332 | -.025 |
| PDE4B | .734 | -.053 | .127 |
| EREG | .731 | .252 | -.023 |
| ATF3 | .719 | .054 | -.069 |
| IL1A | .678 | .166 | .093 |
| PTX3 | .652 | .403 | .077 |
| PTGS2 | .609 | .177 | .195 |
| DUSP2 | .578 | .422 | .008 |
| TNF | .566 | .041 | -.094 |
| ADM | .505 | .344 | -.128 |
| BCL2A1 | .435 | .382 | -.203 |
| **CCL7** | -.046 | .961 | .128 |
| **NAB2** | -.104 | .952 | -.046 |
| **PTPN7** | -.056 | .921 | -.019 |
| CCL2 | -.109 | .895 | .408 |
| STX1A | .057 | .878 | -.175 |
| THBD | -.033 | .801 | -.192 |
| MAFF | .156 | .755 | .183 |
| IRAK2 | .250 | .733 | .015 |
| MAPK6 | .175 | .664 | -.034 |
| DHRS3 | .034 | .637 | -.036 |
| BTG3 | .344 | .605 | .105 |
| SERPINB2 | .314 | .526 | .150 |
| MXD1 | .341 | .519 | .181 |
| IL1R1 | .082 | .485 | -.058 |
| CDC42 | .371 | .447 | -.332 |
| HSPA1A/B | .210 | -.301 | .159 |
| CD9 | .121 | .257 | -.212 |
| **IFIT3** | -.085 | -.027 | .934 |
| **IFI44** | .053 | .168 | .861 |

**Supplementary table 3.** Complete results of the comparison of monocyte gene expression between BD patients and HC. Results are expressed as fold change relative to HC.

| **GENE** | **MEAN** | **95% CI** | | | **P-VALUE** |
| --- | --- | --- | --- | --- | --- |
| **Cluster 1** |  |  | | |  |
| IL1B | 0.72 | -0.76 | - | -0.17 | **<0.001** |
| CCL20 | 0.58 | -1.22 | - | -0.35 | **<0.001** |
| IL6 | 0.76 | -0.71 | - | -0.06 | **.020** |
| TNFAIP3 | 0.79 | -0.48 | - | -0.20 | **<0.001** |
| CXCL2 | 0.66 | -0.92 | - | -0.27 | **<0.001** |
| PDE4B | 0.83 | -0.45 | - | -0.10 | **<0.001** |
| EREG | 0.71 | -0.78 | - | -0.22 | **<0.001** |
| ATF3 | 1.08 | -0.06 | - | 0.28 | .210 |
| IL1A | 0.62 | -1.02 | - | -0.35 | **<0.001** |
| PTX3 | 0.79 | -0.50 | - | -0.16 | **<0.001** |
| PTGS2 | 0.85 | -0.43 | - | -0.03 | **.030** |
| DUSP2 | 0.75 | -0.64 | - | -0.17 | **<0.001** |
| TNF | 0.79 | -0.53 | - | -0.14 | **<0.001** |
| ADM | 0.97 | -0.18 | - | 0.10 | .590 |
| BCL2A1 | 0.84 | -0.46 | - | -0.03 | **.030** |
| **Cluster 2** |  |  |  |  |  |
| CCL7 | 0.93 | -0.62 | - | 0.40 | .670 |
| NAB2 | 0.80 | -0.59 | - | -0.06 | **.020** |
| PTPN7 | 1.02 | -0.11 | - | 0.18 | .660 |
| CCL2 | 1.05 | -0.29 | - | 0.43 | .700 |
| STX1A | 0.97 | -0.37 | - | 0.26 | .750 |
| THBD | 0.95 | -0.26 | - | 0.10 | .380 |
| MAFF | 0.77 | -0.60 | - | -0.14 | **<0.001** |
| IRAK2 | 0.68 | -0.82 | - | -0.29 | **<0.001** |
| MAPK6 | 0.91 | -0.24 | - | -0.03 | **.010** |
| DHRS3 | 0.79 | -0.52 | - | -0.17 | **<0.001** |
| BTG3 | 1.00 | -0.12 | - | 0.13 | .960 |
| SERPINB2 | 0.92 | -0.33 | - | 0.10 | .290 |
| MXD1 | 1.05 | -0.02 | - | 0.17 | .120 |
| IL1R1 | 1.11 | -0.04 | - | 0.34 | .110 |
| CDC42 | 0.97 | -0.17 | - | 0.09 | .560 |
| **Interferon** |  |  |  |  |  |
| IFIT3 | 1.04 | -0.16 | - | 0.26 | .630 |
| IFI44 | 1.01 | -0.15 | - | 0.18 | .860 |
| **Other** |  |  |  |  |  |
| HSPA1A/B | 0.98 | -0.11 | - | 0.07 | .610 |
| CD9 | 1.18 | -0.05 | - | 0.52 | .100 |
| **Hepatocyte growth factor** | 1.10 | 0.04 | - | 0.23 | **<0.001** |
| **Glucocorticoïd receptors** |  |  |  |  |  |
| GRα | 1.03 | 0.00 | - | 0.10 | **.040** |
| GRβ | 0.97 | -0.20 | - | 0.12 | .630 |
